# Flexible Non-contact Electrodes for Wearable Biosensors System on Electrocardiogram Monitoring in Motion

**DOI:** 10.3389/fnins.2022.900146

**Published:** 2022-06-07

**Authors:** Xin Wang, Shuting Liu, Mingxing Zhu, Yuchao He, Zhilong Wei, Yingying Wang, Yangjie Xu, Hongguang Pan, Weimin Huang, Shixiong Chen, Guanglin Li

**Affiliations:** ^1^CAS Key Laboratory of Human-Machine Intelligence-Synergy Systems, Shenzhen Institute of Advanced Technology, Chinese Academy of Sciences, Shenzhen, China; ^2^Shenzhen College of Advanced Technology, University of Chinese Academy of Sciences, Shenzhen, China; ^3^Guangdong-Hong Kong-Macao Joint Laboratory of Human-Machine Intelligence-Synergy Systems, Shenzhen Institute of Advanced Technology, Chinese Academy of Sciences, Shenzhen, China; ^4^Department of Informatics, Technical University of Munich, Munich, Germany; ^5^School of Electronics and Information Engineering, Harbin Institute of Technology, Shenzhen, China; ^6^Interdisciplinary Centre for Security, Reliability and Trust, University of Luxembourg, Luxembourg, Luxembourg; ^7^Department of Otolaryngology, Shenzhen Children’s Hospital, Shenzhen, China; ^8^Department of Neonatology, Shenzhen Children’s Hospital, Shenzhen, China

**Keywords:** wearable biosensors system, flexible non-contact electrode, electrocardiogram, monitoring, motion

## Abstract

Electrocardiogram (ECG) is a critical physiological indicator that contains abundant information about human heart activities. However, it is a kind of weak low-frequency signal, which is easy to be interfered by various noises. Therefore, wearable biosensors (WBS) technique is introduced to overcome this challenge. A flexible non-contact electrode is proposed for wearable biosensors (WBS) system, which is made up of flexible printed circuits materials, and can monitor the ECG signals during exercise for a long time. It uses the principle of capacitive coupling to obtain high-quality signals, and reduces the impact of external noise through active shielding; The results showed that the proposed non-contact electrode was equivalent to a medical wet electrode. The correlation coefficient was as high as 99.70 ± 0.30% when the subject was resting, while it was as high as 97.53 ± 1.80% during exercise. High-quality ECG could still be collected at subjects walking at 7 km/h. This study suggested that the proposed flexible non-contact electrode would be a potential tool for wearable biosensors for medical application on long-term monitoring of patients’ health and provide athletes with physiological signal measurements.

## Introduction

Physiological signals are widely used in medical applications and health monitoring, and they serve as the objective indicators closely related to human health (Le et al., 2010). For example, the ECG signals that contain abundant information of the heart activities provide useful information for disease prevention (Zhang et al., 1997). And it is a critical physiological indicator for screening cardiovascular diseases and evaluating heart or cardiovascular functions. Therefore, the standard wet silver chloride (Ag/AgCl) electrodes as the most commonly used biopotential electrodes for measuring electrocardiogram (ECG) signals is found in hospital-based medical diagnostic and home health monitoring systems (Fernandez and Pallas-Areny, 2000). However, the ECG signal is a low-frequency weak signal and is susceptible to various noise interferences during acquisition (Song and Yu, 2010). For example, Muscle cells tremble as they contract, producing high-frequency EMG signals, which have a high frequency and a low amplitude (Deng et al., 2000). This EMG signal will cause interference to the original signal when collecting ECG. The affected ECG signal appears as a small glitch in the time domain. In addition, breathing can also affect the acquisition of signals. While breathing, people are accompanied by small fluctuations in the body, producing lower frequency interference signals (Wu et al., 2014). The baseline of a normal ECG signal is a straight line, while the baseline of the interfered signal is no longer a straight line, often referred to as baseline drift (Chouhan and Mehta, 2007; Yu et al., 2014). Moreover, the impact of motion on signal acquisition is more obvious (Garcia-Casado et al., 2006; Lee et al., 2014; Torfs et al., 2014; Pei et al., 2016). This kind of interference is uncontrollable and will cause a large change in the signal. Therefore, general ECG acquisition requires the subject to remain as static as possible (Liu et al., 2018). In the state of motion, the electrode and the human skin will be distorted and displaced. Friction can cause changes in the impedance between the skin and the electrodes, causing some interference during the entire process.

Due to the special occupation of athletes, higher requirements are placed on the electrodes. Currently used in the clinic is a standard wet electrode, which reduces the impedance between the skin and the electrode through a conductive gel to achieve high-quality ECG (Li et al., 2018). However, there are still lots of problems to be solved. For high-quality ECG monitoring, researchers did their efforts on the Wearable Biosensors, most of which are dry electrodes that do not require conductive gels (Dozio et al., 2007; Assambo and Burke, 2009; Zhang et al., 2015; Joutsen et al., 2017). Kim et al. (2016) proposed a 1D-2D hybrid carbon nanocomposites-based ECG electrode and recorded ECG with three movements, namely wrist curl, squat, and writing. Noh et al. (2016) proposed a novel conductive carbon black and PDMS ECG electrode which could achieve ECG recording with water exposed conditions. Asadi et al. (2021) realized continuous medical ECG monitoring by constructing a graphene elastomer electrode. Even though plenty of WBSs were proposed, none of the existing methods met the requirement on ECG monitoring in motion. Besides, the dry electrodes without conductive gel would have the impedance between electrodes and skin rather high, which makes the recorded signal be easily affected by the body motion of the subjects (Pei et al., 2016). Non-contact electrodes, which can make measurements through clothing without any contact between the electrode and the skin, is essential for building user-friendly WBS networks for physiological recording during motion (Lee et al., 2013). Up to date, most of the non-contact electrodes are rigid and sensitive to motion (Chi et al., 2009, 2010, 2011, 2013), not benefiting for long-term monitoring. Therefore, monitoring ECG signals during motion is definitely a challenge.

In this study, a flexible non-contact electrode for WBS was proposed based on the principle of capacitive coupling for physiological signal acquisition. The electrode was built with flexible printed circuits (FPC) materials and could be bent to ensure better capacitive coupling with the skin (Yi et al., 2018; Liu et al., 2019). Although the motion had a great influence, the proposed non-contact electrode could acquire high-quality ECG compared with the wet electrode. In this study, we first studied the effects of the layers of the insulation materials on flexible non-contact electrode-based ECG quality compared with the gold standard method. Then, we further studied on the effect of the size of the flexible non-contact electrode on the ECG quality. After that, we investigated the performance of flexible non-contact electrodes on ECG recording at different walking speeds.

## Materials and Methods

### Subjects

Three male subjects aged from 20 to 25 years old were recruited in this study. The subjects had normal cardiac function, normal muscle function, and no cognitive impairments. The experimental procedures were clearly explained to the subjects, and the data collection was carried out in an ordinary laboratory environment without any electromagnetic shielding. All the experimental protocols were approved by the Institutional Review Board (IRB) of the Shenzhen Institutes of Advanced Technology, Chinese Academy of Sciences. (SIAT-IRB-190615-H0352).

### The Experimental Scheme

**FIGURE 1:**
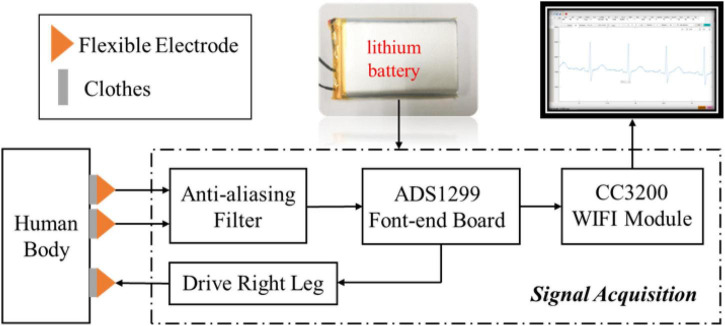
The overall design block diagram of the non-contact ECG signal acquisition system.

**FIGURE 2:**
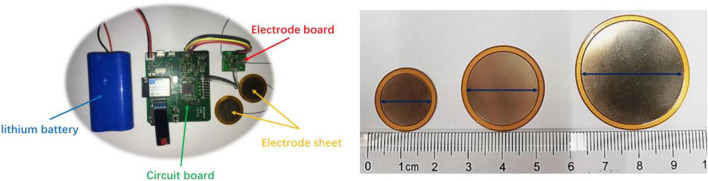
The photos of the ECG signal acquisition system and flexible non-contact electrodes with different sizes [small (the diameter is 1.5 cm), medium (the diameter is 2.1 cm), and large (the diameter is 3.0 cm)] electrode.

The overall design block diagram of the system was shown in Figure 1. Two non-contact electrode sheets and one driven right leg (DRL) electrode were used to collect physiological electrical signals (Guermandi et al., 2008; Kim and Park, 2008; Guerrero and Spinelli, 2017). The physiological signal monitoring of non-contact electrodes mainly utilizes the principle of capacitive coupling. The non-contact electrode sheet and the skin corresponds to both the conductive surface of the capacitor, while the clothing corresponds to the insulating dielectric-filled in the middle of the capacitor. The signal coupled into the circuit was pre-processed by an anti-aliasing filter, and then entered the ADS1299 (Texas Instruments, Dallas, Texas, United States), a front-end board, for signal acquisition and conversion from analog signals into high-resolution digital signals. The data acquisition process was controlled by the wireless CC3200 MCU (Texas Instruments, Dallas, Texas, United States) and the data from ADS1299 was streamed through high-speed Wi-Fi to the PC, where a Matlab (Mathworks Inc., United States) GUI was ready to display the real-time waveform and stored the raw data for offline analyses. The physiological electrical signal acquisition system was shown in Figure 2, including a circuit board, electrode board, and electrode sheets. The electrode sheet was of round shape and composed of a double-layer flexible printed circuit board (FPCB), which could be bent according to the skin curve. The bottom layer was completely filled with copper to prevent the sensing plate from external interferences; the top layer was composed of one circular copper-filled non-contact capacitive sensing plate and one concentric outer shielding ring connected with the outer ring at the bottom to improve the shielding outcomes (Muneer, 2014; Chen et al., 2017; Jiang et al., 2017). The flexible non-contact electrode was connected with the electrode board using a soft shielded cable, whose inner wire was connected with the top sensing plate and the outer shield was connected with the shielding layer. The ADS1299 and CC3200 WIFI modules were soldered on the circuit board. In addition, the lithium battery was used for the power supply to reduce power frequency interference.

## Results

### The Effects of Insulation Layers and Electrode Size

**FIGURE 3:**
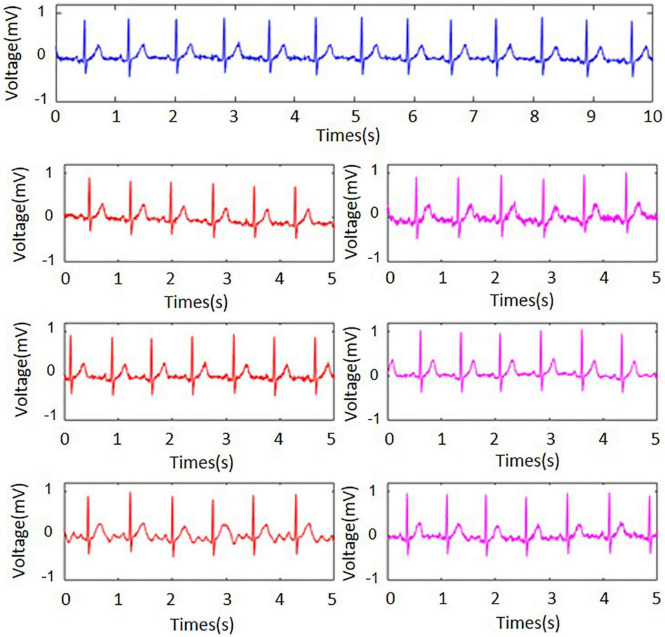
Static electrocardiographic signals collected under different electrodes (blue for wet electrode, red and pink for flexible non-contact electrode) and under different thicknesses of canvas (red for a layer of canvas and pink for three layers). The second, third, and fourth rows used small, medium, and large electrodes, respectively.

To verify the performance of the proposed electrodes, the standard wet electrodes were used as a reference, which was shown by the blue line in Figure 3. The red signals were the ECG signals collected by our electrodes when a layer of canvas was used as the insulating material, while the pink signal was acquired when three layers of canvas were used as the insulating material. All signals were collected synchronously while the subject remained relaxed stationary position. It was clear that the ECG signal quality was high, the baseline was very thin and stable and P-wave, T-wave, and QRS waves could be clearly observed. However, as the number of interlayer materials increased, the signal quality tended to decline. In the figure, the baseline was widened and the signal contains burrs, but it did not evidently influence the quality. The second, third, and fourth rows in the figure used small (the diameter is 1.5 cm), medium (the diameter is 2.1 cm), and large (the diameter is 3.0 cm) electrode sheets, respectively. As the size of the electrode sheets increased, the amplitude of the ECG signal increased a little bit.

Among them, the medium electrode signal quality was the best, and the electrode sheet was too large or too small would have an impact on the signal quality. If the electrode piece was too small, it would cause a less effective signal to be transmitted, affecting the signal quality, while if the electrode piece was too large, and in the case of non-contact, the noise generated by the friction would be mixed into the signal, reducing the signal-to-noise ratio. In all, our non-contact electrodes could acquire high-quality ECG signals which were highly similar to the standard wet electrode signal, when the subject was quiet.

### Electrocardiogram Collected During Motion

**FIGURE 4:**
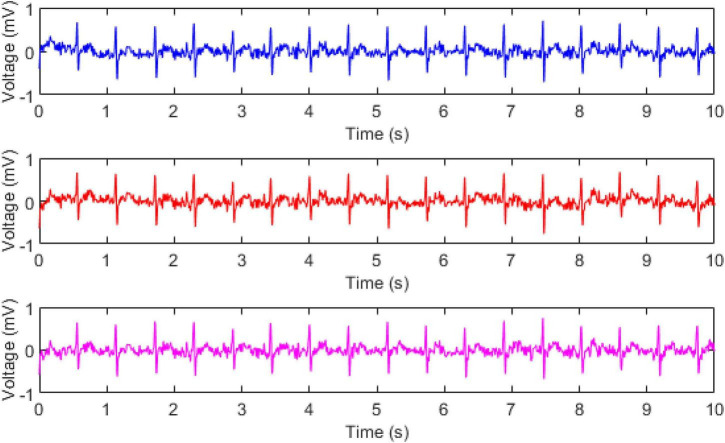
The ECG recorded by wet electrode (blue), proposed electrode (red), dry electrode (pink) at the speed of 2 km/h.

The flexible non-contact electrodes could not only capture signals when the subject was quiet but also acquired signals when they were in motion. When the subject was exercising at a speed of 2 km/h on the treadmill, we selected the medium electrode piece as the non-contact electrode for signal monitoring. At the same time, we used the standard wet electrode and the dry electrode for simultaneous monitoring, and the positions of the electrode pieces were as close as possible. The original ECG time-domain waveforms obtained by the three different electrodes including the wet, flexible non-contact, and dry electrodes, were shown in Figure 4. It could be observed that the signals collected by the three electrodes were highly similar. Therefore, although the subject was in motion, the proposed non-contact electrodes, especially the flexible one, could achieve a comparable quality of signals with the standard wet electrodes in ECG measurements.

### Electrocardiogram Collected With Different Walking Speeds

**FIGURE 5:**
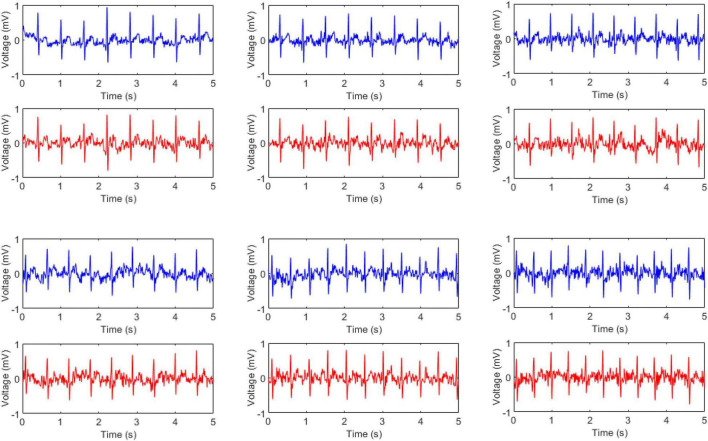
The ECG signals recorded by wet (blue) and flexible non-contact electrode (red) under different walking speeds (from left to right and from up to down, they were 2, 3, 4, 5, 6, and 7 km/h, respectively).

Next, we used the flexible non-contact electrode to further investigate the ECG monitoring during exercise. Subjects were kept at a constant speed from 2 to 7 km/h on the treadmill. At the same time, we used non-contact electrodes and standard wet electrodes to monitor ECG signals, as shown in Figure 5. It could be observed from the figure that as the speed of the subject increased, the number of R waves detected during the same time increased, which was in line with the mechanism of human motion. Besides, as the speed increased, the signal quality of both the non-contact electrode and the wet electrode decreased, and the T wave and the P wave were gradually unclear, but the R wave could still be clearly detected.

### Mathematical Statistics Analysis

**FIGURE 6:**
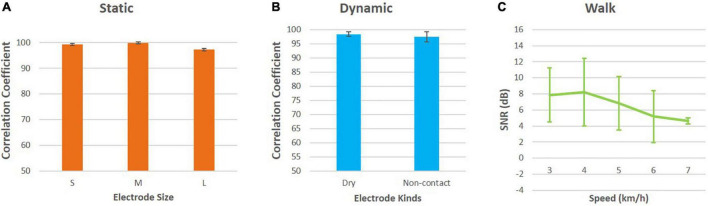
Mathematical statistics analysis: **(A)** Correlation coefficients between electrodes of different sizes and wet electrodes under static conditions; **(B)** correlation coefficients between flexible non-contact electrodes, flexible dry electrodes and wet electrodes under motion; **(C)** SNR at different walking speeds.

When the subject remained stationary, the small, medium, and large electrodes all had a high correlation coefficient with the wet electrode, as shown in Figure 6, indicating that the perfectly designed flexible electrode can perform ECG monitoring in a non-contact state. When studying the effects of insulation layers and electrode size, the ECG signals from the wet and non-contact electrodes were compared and analyzed by calculating the average correlation coefficient. An average correlation coefficient of 99.28 ± 0.42, 99.70 ± 0.30, and 97.18 ± 0.52% was calculated for the small, medium and large ECG electrodes, respectively when compared to the wet Ag/AgCl electrode. Therefore, the size of the electrode would bring about a difference in the correlation coefficient, which meant that the size of the electrode would affect the signal. For example, the medium-sized electrode had the best signal. When the electrode pad area became smaller, the effective signal would decrease; when the electrode pad area became larger, the noise entering the circuit would also increase. When considering the condition in motion, taking the standard wet electrode as the gold standard, the correlation coefficient between the non-contact electrode and the wet electrode was as high as 97.53 ± 1.80%, and the correlation coefficient between the dry electrode and the wet electrode was as high as 98.43 ± 0.77%. Moreover, we calculated the SNR of the ECG acquired by the proposed electrode under different walking speeds, the result showed that with the increment of the speeds, the quality of the ECG could maintain reliable.

## Discussion

### Effects of Electrode Size

The non-contact electrode uses the principle of capacitive coupling (Wang et al., 2015; Li and Sun, 2017). According to the formula of the capacitance, the capacitance increases as the area of the electrode sheet increases, and decreases as the distance between the electrode sheet and the skin increases. However, according to actual experiments, the quality and strength of effective signals do not increase with the increase in electrode pad size. In this experiment, we used non-contact electrodes with diameters of 1.5, 2.1, and 3 cm, with an area ratio of 1:1.4:2, respectively. As the area of the electrode sheets increased, the amplitude of the electrocardiographic signal appeared to become larger, but not significant. At the same time, we used the method of calculating the correlation coefficient to evaluate the ECG signal quality compared with the standard wet electrode. An average correlation coefficient of 99.28 ± 0.42, 99.70 ± 0.30, and 97.18 ± 0.52% was calculated for the small, medium and large ECG electrodes, respectively when compared to the wet Ag/AgCl electrode. The results demonstrated that the proposed electrode performed as better as the gold standard method does. Besides, we found that the signal quality of the medium electrode was the best, so in the non-contact acquisition, the quality of the ECG signal and the area of the electrode pad were not simply proportional. It was possible that as the area of the electrode sheet became larger, the effective signal was increased, but at the same time, noise interference was also introduced.

### Effects of Clothing Thickness

Since the non-contact electrode is based on the capacitive coupling between the electrode plate and the skin surface, the effective capacitance is proportional to the area of the electrode plate and the dielectric constant of the insulating material while inversely proportional to the distance between the electrode and the skin. As the thickness of the insulating layer increases, the signal quality decreases. In theory, the further the electrode is from the source, the smaller the coupling signal (Liu et al., 2019). In this experiment, we used two thicknesses of clothing for monitoring and both obtained an effective ECG signal, namely one layer and three layers. The result showed that thin clothes had less noise interference than thick clothes. It was consistent with the principle of capacitive coupling. As the layers of the insulation materials increase, the distance between the electrode and the skin increase, which finally causes the decrease of the effective capacitance and therefore leads to the deterioration of the ECG quality. This result was consistent with the existing research that had been reported (Chi et al., 2009; Mathias et al., 2015; Wang et al., 2019). And in our study, for three layers of the insulating material, the ECG signals still had recognizable ECG characteristics, which could be used for further analysis.

### Effects of Walking Speed

We not only performed ECG signal acquisition when the subject was at rest but also monitored the subject at constant speeds ranging from 2 to 7 km/h with the increment of 1 km/h. The results showed that as the speed increased, the ECG cycle became shorter, which was consistent with the mechanism of human motion that the heart would beat faster when the human being did exercise. As the speed increased, the wet-electrode and our electrode signal quality tended to decrease, mainly affected by myoelectric signals and noise from electrodes friction and breathing. When the subject was walking, muscle cells tremble as they contract, producing high-frequency EMG signals, which had a high frequency and a low amplitude. This EMG signal would cause interference to the original signal when collecting ECG. The affected ECG signal appeared as a small glitch in the time domain. With the increase of speed, the EMG signal became stronger and the interference to the ECG became larger. During the exercise, the electrodes and skin would rub against the clothes and cause relative displacement. At this time, a large amount of motion artifact noise was generated. It was no doubt that motion artifacts had a great impact on the signal. As the speed increased, the motion artifacts also increased, therefore, leading to the quality of the ECG signal decreasing. In addition, breathing could also affect the acquisition of signals. While breathing, people were accompanied by small fluctuations in the body, producing lower frequency interference signals. The baseline of a normal ECG signal was a straight line, while the baseline of the interfered signal was no longer a straight line, often referred to as baseline drift. The deterioration of signal quality was mainly manifested by the thickening of the baseline and the unclear T and P waves. However, the QRS waveform could be clearly seen in this study. Overall, our electrodes were resistant to noise interference during motion monitoring, with good signal quality and a correlation coefficient of 97.53 ± 1.80% with standard wet electrodes. The SNR of ECG in 3, 4, 5, 6, and 7 km/h were 7.87 ± 3.36, 8.23 ± 4.22, 6.83 ± 3.33, 5.20 ± 3.24, and 4.64 ± 0.40 dB, respectively. This result suggested that the proposed electrode could achieve high quality ECG recording even in motion. In the future, we would like to recruit more subjects with different sexualities and weights to further evaluate the proposed electrode.

### Noises in Electrocardiogram

In common practices, the recorded ECG signals are easily contaminated by noises such as powerline interferences, electrode displacement, electrode lead jitter, EMG noises and so on. It could be observed from Figures 3–5 that the recorded ECG signals for our proposed non-contact electrode did now show significant contamination by the abovementioned noises. Such anti-noise capacity was achieved by the following technologies of our hardware and experimental protocol: (1) the active shielding technology (Jiang et al., 2018) in which all the frontend pathway of the non-contact electrode was shielded by a buffered voltage of the same level as the inner-wire signal, so that the capacitive coupling to the mains and to the ground could almost be eliminated, leading to extraordinary attenuation of the powerline interferences. With the assistance of the same active shielding technology, the interferences introduced by electrode lead jitter during body movements were also eliminated, which simplifies the subsequent signal processing and analysis tasks. (2) the proposed non-contact electrode was quite flexible so that it could bend freely according to the local curvation of the skin. In this way, it was very difficult for the electrode to move after it was fixed, even when the subject was running. The noises introduced by electrode displacement were prevented from degrading the ECG signal quality as a result. (3) in the experimental protocol, the electrodes were placed in the forearm positions where there were few muscles underneath. The subjects were also asked to avoid making large forearm muscle contractions when running. In this way, there were no significant EMG noise interferences in the ECG recording of this study. The data acquisition system of this study with flexible non-contact electrode could be a great tool to measure ECG signals with robust performance in noise immunity.

## Conclusion

In this study, we proposed a wearable device for dynamic ECG monitoring, whose electrodes were flexible and non-contact, which was very friendly to subjects. When developing WBS devices, consideration should be given to user comfort, stability of long-term monitoring, and reliability of acquired signals. To enhance user comfort, we designed a flexible capacitive electrode that could bend along the curvature of the body surface. This capacitive coupling means that the ECG signal can be monitored while the subject is wearing the clothes, which avoids the possibility of allergy when using a wet electrode. We designed the electrode shielding ring to reduce noise interference and monitor the motion of the motion center. The results showed that the proposed electrodes could effectively resist the interference of noise. The ECG signal collected at rest reached the correlation coefficient of 99.70 ± 0.30% compared with the standard wet electrode. While, in the motion state, the correlation coefficient of the signal was as high as 97.53 ± 1.80% compared with the wet electrodes and dry electrodes. We verified the performance of the electrode by walking on a treadmill at a speed of up to 7 km/h. The results confirmed the feasibility of the developed system in daily life ECG monitoring, and suggested that the proposed flexible non-contact electrode would be a potential candidate for WBS systems on long-term healthcare monitoring for patients and physiological signal measurements for athletes.

## Data Availability Statement

The data used to support the findings of this study are available on reasonable request from the corresponding authors.

## Ethics Statement

The studies involving human participants were reviewed and approved by the Institutional Review Board of Shenzhen Institutes of Advanced Technology, Chinese Academy of Sciences (SIAT-IRB-190615-H0352). The patients/participants provided their written informed consent to participate in this study.

## Author Contributions

SC: conceptualization and supervision. SL, XW, HP, and WH: data curation. SC, GL, and MZ: funding acquisition. SL, XW, and YW: methodology. XW, YH, and ZW: validation. XW and YX: visualization. SL and XW: writing—original draft. XW and SC: writing—review and editing. All authors contributed to the article and approved the submitted version.

## Conflict of Interest

The authors declare that the research was conducted in the absence of any commercial or financial relationships that could be construed as a potential conflict of interest.

## Publisher’s Note

All claims expressed in this article are solely those of the authors and do not necessarily represent those of their affiliated organizations, or those of the publisher, the editors and the reviewers. Any product that may be evaluated in this article, or claim that may be made by its manufacturer, is not guaranteed or endorsed by the publisher.
